# Tumor escape and progression of HER-2/neu negative breast cancer under immune pressure

**DOI:** 10.1186/1479-5876-9-35

**Published:** 2011-03-31

**Authors:** Maciej Kmieciak, Kyle K Payne, Michael O Idowu, Margaret M Grimes, Laura Graham, Maria-Libera Ascierto, Ena Wang, Xiang-Yang Wang, Harry D Bear, Masoud H Manjili

**Affiliations:** 1 Department of Microbiology & Immunology, Virginia Commonwealth University Massey Cancer Center, 401 College Street, Richmond VA 23298, USA; 2 Department of Pathology, Virginia Commonwealth University Massey Cancer Center, 1200 E. Marshall Street, Richmond VA 980662, USA; 3 Department of Surgery, Virginia Commonwealth University Massey Cancer Center, 1200 E. Broad Street, Richmond VA 980011, USA; 4 Infectious Disease and Immunogenetics Section (IDIS), Department of Transfusion Medicine, Clinical Center and Center for Human Immunology (CHI), National Institutes of Health, 10 Center Drive, Bethesda, MD 20892, USA; 5 Department of Human and Molecular Genetics, Virginia Commonwealth University Massey Cancer Center, 401 College Street, Richmond VA 23298, USA

## Abstract

**Background:**

Emerging data from pre-clinical and clinical studies suggest that HER-2/neu-specific T cell responses could induce HER-2/neu antigen loss in the tumor cells. These data suggest that patients with HER-2/neu negative breast cancer might have had HER-2/neu positive premalignant lesions in the past that progressed to HER-2/neu negative breast cancer under HER-2/neu-specific immune pressure.

**Methods:**

We conducted a pilot study in patients with HER-2/neu positive and HER-2/neu negative breast cancers as well as a patient with ductal carcinoma in situ (DCIS). HER-2/neu expression was determined by FISH. HER-2/neu-specific T cell responses were determined by using IFN-γ ELISA. Expression of IFN-γ Rα in the tumors was determined by immunohistochemistry analysis of paraffin-embedded tissues.

**Results:**

We determined that majority of (10 of 12) patients with HER-2/neu negative breast cancer had HER-2/neu-specific IFN-γ producing T cell responses which was stronger than those in patients with HER-2/neu positive tumors. Such immune responses were associated with nuclear translocation of IFN-γ Rα in their tumor cells. Patient with DCIS also showed HER-2/neu-specific T cell responses.

**Conclusion:**

These data suggest that conducting retrospective studies in patients with HER-2/neu negative breast cancers and prospective studies in patients with HER-2/neu positive DCIS can determine whether HER-2/neu negative invasive carcinomas arise from HER-2/neu positive DCIS under the immune pressure.

## Introduction

In recent years, there has been emerging evidence from both pre-clinical and clinical studies, including our own, which challenge the one-sided view of the role of IFN-γ producing T cells in protecting the host against cancers. For example, IFN-γ was shown to promote immune editing and subsequent tumor escape in the CT26 colon carcinoma by down-regulation of the expression of gp70 immunogenic tumor antigen at the mRNA levels [1]. Recently, it was demonstrated that inhibiting expression of IFN-γ receptor (R) by forcing expression of the dominant negative IFN-γ R reduced the ability of renal carcinoma cells to metastasize [2]. We have also reported that immunotherapy of rat neu expressing breast cancer elicits neu-specific IFN-γ producing CD8+ T cells that in turn facilitate breast cancer recurrence of neu Antigen Negative Variant (ANV) tumors following initial rejection of the neu positive Mouse Mammary Carcinoma (MMC) tumor cells in immunocompetent mice [3,4]. The tumor antigen loss was due to hypermethylation of the neu promoter and loss of neu both at mRNA and protein levels [3,5], resulting in escape of the tumor from further neu-specific immune responses. On the clinical front, elevated serum levels of IFN-γ in uveal melanoma patients correlate with the spread of metastasis and represent a negative prognostic marker [6]. It was recently shown that HER-2/neu-targeted vaccination of patients who had HER-2/neu+ ductal carcinoma *in situ *(DCIS) elicited HER-2/neu-specific IFN-γ producing CD8+ T cell responses which resulted in HER-2/neu antigen loss [7]. Although the authors considered this HER-2/neu loss a positive outcome of the immune response, no follow-up studies have been performed to determine whether patients with HER-2/neu loss in their DCIS tumors might end up with recurrence of invasive HER-2/neu positive or negative breast cancers. Several other correlative or *in vitro *studies suggest potentially negative effects of some immune responses in breast cancer. Matkowski and Sheu both showed in cohorts of 88 and 24 patients with operable breast cancer, respectively, that relapse or disease progression was associated with strong CD8+ T cell infiltration [8,9]. Interestingly, it was reported that HER-2/neu+ human prostate tumor cell lines, DU145 and PC-3, that responded to IFN-γ (because of the expression of IFN-γ Rα), showed down-regulation of HER-2/neu expression whereas another prostate tumor cell line, LNCaP, that failed to respond to IFN-γ did not show any change in the expression of HER-2/neu [10]. Such failure of the LNCaP to respond to IFN-γ was later shown to be due to the lack of JAK1 expression [11]. These findings prompted us to determine whether HER-2/neu-specific IFN-γ producing T cell responses may be associated with HER-2/neu loss and progression to HER-2/neu negative breast carcinoma. To test this hypothesis, we conducted pilot studies in patients with HER-2/neu positive and HER-2/neu negative breast carcinoma to determine whether patients with HER-2/neu negative tumors had HER-2/neu-specific T cell responses that had been induced by HER-2/neu positive pre-malignant lesions in the past.

## Materials and methods

### Patient specimens

Formalin-fixed paraffin-embedded tumor blocks were prepared from 15 patients with breast cancer among which 12 patients had HER-2/neu negative tumors and three patients had HER-2/neu positive tumors, as determined by fluorescence in situ hybridization (FISH). We also included two samples from a patient with ductal carcinoma in situ (DCIS). Peripheral blood mononuclear cells (PBMCs) and sera were also obtained from these patients and used for *in vitro *studies. This study was conducted under Institutional Review Board (IRB) protocol# HM10920 at Virginia Commonwealth University. All patients had the capacity to give informed consent to participate in this research.

### IFN-γ ELISA

PBMCs were harvested from the blood of invasive breast cancer patients (n = 15) and two samples from a patient with DCIS. After Ficoll density gradient separation, PBMCs were cultured at 37°C for 2 hr, adherent cells were used for the generation of monocyte-derived DCs in the presence of GM-CSF (100 ng/ml) and IL-4 (50 ng/ml), as previously described by our group [12]. Floater cells were maintained with IL-2 (40 U/ml/10^6 ^cells) in complete medium for 6-7 days until autologous DCs became available. The IL-2 maintained lymphocytes were then cultured with autologous DCs (4:1) in the presence or absence of recombinant HER-2/neu (100 μg/ml) or LPS (10 μg/ml). After 24 hs, supernatants were collected and subjected to IFN-γ ELISA. We used a Human IFN-γ ELISA set according to the manufacture's protocol (BD Pharmingen).

### Recombinant HER-2/neu protein

The SK-BR-3 breast tumor line that overexpresses HER-2/neu was used to prepare the cDNA. Extracellular domain (ECD) and intracellular domain (ICD) of HER-2/neu were amplified using specific primers. ECD-forward: 5' AAA CTC GAG ATG GAG CTG GCG GCC TTG T 3' and reverse: 5' CTT AAG CTT CGT CAG AGG GCT GGC TCT CT 3'; ICD-forward: 5' AAA CTC GAG AAG CGA CGG CAG CAG AAG AT 3' and reverse: 5' CTT AAG CTT TCA CAC TGG CAC GTC CAG 3'. The orientation and integrity of inserted sequence were screened by detailed restriction analysis and sequencing. DNA encoding the ECD (aa 1-695) or ICD (aa 692-1256) were ligated into the expression vector pRSET (Invitrogen). The BL21 (DE3) pLysS strain of *E. coli *was used for the expression the proteins. Proteins were purified under denaturing condition using Invitrogen ProBond Purification System. The ECD and ICD proteins were then dialyzed using 10 mM Tris, pH 8.0, and 20 mM Tris pH 9.0, respectively. Purity of the proteins was above 90% as determined by SDS-PAGE.

### Immunohistochemistry (IHC)

In order to determine the status of IFN-γ Rα expression in the tumors IHC was performed using Dako automated immunostainer (Dako, Carpinteria, CA). We used anti-human IFN-γ Rα antibody (Santa Cruz Biotechnology Inc., Santa Cruz, CA). The antigen retrieval was achieved using a rice steamer. In order to circumvent the endogenous biotin activity, we used Dako Envision Dual Link System-HRP (Dako, Capinteria CA) in a two-step IHC technique, based on HRP labeled polymer which is conjugated with secondary antibodies. The labeled polymer does not contain avidin or biotin, thereby avoiding the non specific endogenous avidin-biotin activity in the sections. Positive and negative controls stained appropriately.

### Statistical analysis

Statistical comparisons between groups were made using an unpaired Student's *t *test with P < 0.05 being statistically significant.

## Results

### Detection of HER-2/neu-specific IFN-γ positive T cell responses in HER-2/neu negative breast cancer patients

**Figure 1:**
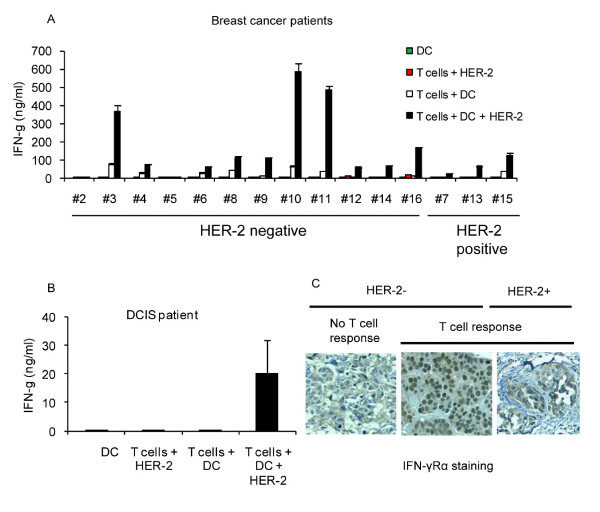
**HER-2/neu-specific T cell responses in patients with HER-2/neu negative or positive breast cancers**. Peripheral blood T cells were isolated from patients with HER-2/neu positive (patients #7, 13, 14) or HER-2/neu negative (all other patients) breast cancers (stage I-III) (A) and two samples from DCIS patient (B). T cells were then co-cultured with or without DCs in a 2:1 ratios in the presence or absence of recombinant human HER-2/neu (intracellular domain + extracellular domain: ECD+ICD, 100 μg/ml) for 24 hrs. Supernatants were collected and subjected to IFN-γ ELISA. C) Representative IHC staining (200× magnification) of tumor lesions of patients with HER-2/neu negative (-) and HER-2/neu positive (+) tumors who had HER-2/neu-specific T cell responses or no T cell responses.

Of 12 patients with HER-2/neu negative breast cancers (stages I-IIIA) ten patients showed HER-2/neu-specific IFN-γ producing T cell responses (Figure 1A). All three patients with HER-2/neu positive breast cancer also showed HER-2/neu-specific T cell responses. As shown in Figure 1A, patients with HER-2/neu positive tumors showed significantly lower IFN-γ responses to HER-2/neu compared to HER-2/neu negative patients (HER-2/neu positive: 22-120 ng/ml, HER-2/neu negative: 61-600 ng/ml, *P *= 0.012). These responses were detected in the PBMC of patients prior to any treatment. Samples from a DCIS patient also showed HER-2/neu-specific T cell responses (Figure 1B). No ECD-specific IgG antibody response was detected in the sera of these patients (data not shown). Patient characteristics are shown in Table 1.

**Table 1:** Patients' characteristics

Patients	Stage of tumor	HER-2 status
#2	I	-

#3	I	-

#4	IIA	-

#5	IIB	-

#6	I	-

#8	IIIA	-

#9	IIA	-

#10	IIA	-

#11	IIA	-

#12	I	-

#14	I	-

#16	I	-

#7	IIA	+

#13	I	+

#15	II	+

### Nuclear translocation of IFN-γ Rα in the tumors of patients with HER-2/neu-specific T cell responses

Since nuclear translocation of IFN-γ Rα is a consequence of IFN-γ signaling in the tumor cells, we performed IHC analysis of the tumor lesions of the patients using anti-human IFN-γ Rα antibody. As can be seen in Figure 1C, patients with HER-2/neu negative tumors and who had strong HER-2/neu-specific T cell responses showed an intense and uniform nuclear staining for IFN-γ Rα in their tumors while those with no T cell responses showed weak cytosolic staining for IFN-γ Rα in their tumors. Patients with low levels of HER-2/neu-specific IFN-γ production (HER-2/neu positive or HER-2/neu negative tumors) showed weak and scattered nuclear staining along with cytosolic staining for IFN-γ Rα in their tumors.

## Discussion

If HER-2/neu-specific IFN-γ producing T cells are involved in HER-2/neu loss and tumor recurrence, we might be able to detect such immune responses in patients with HER-2/neu negative breast cancer, who might have had undetectable HER-2/neu positive premalignant tumors in the past, that had lost HER-2/neu expression and progressed to invasive carcinoma under the immune pressure. The fact that 55-75% of patients with premalignant DCIS overexpress HER-2/neu in their tumor lesions and 75% of breast cancers are HER-2/neu negative would suggest the progression of HER-2/neu positive DCIS to HER-2/neu negative breast cancer is only in the tumor clones that express IFN-γ Rα. We have already shown that T cell-mediated tumor antigen loss was due to hypermethylation of the neu promoter and loss of neu both at mRNA and protein levels [3,5]. It is likely that DNA methylation may also impact HER-2/neu gene amplification, as suggested by others [13]. We performed a pilot study accruing 12 breast cancer patients with HER-2/neu negative tumors (HER-2/neu status: FISH negative) and three breast cancer patients with HER-2/neu positive tumors (FISH positive). We detected HER-2/neu-specific IFN-γ producing T cell responses in PBMC of patients with HER-2/neu negative cancers (10 out of 12 patients). Interestingly, there was a direct correlation between the HER-2/neu-specific T cell responses and nuclear localization of IFN-γ Rα in the tumors, as an indication of IFN-γ responses at the tumor site [14]. A higher IFN-γ production in a majority of patients with HER-2/neu negative tumors compared to those with HER-2/neu positive tumors suggests the presence of high affinity T cells in the former. Weak T cell responses were associated with weak and scattered nuclear and cytosolic IFN-γ Rα while stronger T cell responses in patients with HER-2/neu- tumors was associated with an intense and uniform nuclear IFN-γ Rα. Our findings suggest that the increased rate of HER-2/neu positive DCIS compared with breast cancer may reflect the loss of HER-2/neu during tumorigenesis in premalignant cells where IFN-γ signaling pathway is active. This possibility is also supported by a number of observations reported by other groups. For instance, induction of HER-2/neu-specific IFN-γ producing T cell responses in patients with DCIS resulted in loss of HER-2/neu expression [7]. It was also reported that overexpression of HER-2/neu in DCIS lesions predicts the presence of invasive foci in patients with DCIS [15]. Others also have suggested that the low frequency of HER-2/neu expression (20-25%) in invasive breast cancer implies that HER-2/neu loss is an epiphenomenon of disease progression [16].

## Conclusions

The results of this study emphasize a potentially critical role for an inflammatory type of anti-tumor immune responses [17,18] such as IFN-γ which can facilitate tumor antigen loss and relapse of more invasive tumors. Presence of HER-2/neu-specific T cell responses in DCIS patients who have IFN-γ Rα positive lesions may suggest that these patients are at risk of developing HER-2/neu negative breast cancer. This needs to be determined by conducting prospective studies in patients with DCIS.

## Competing interests

The authors declare that they have no competing interests.

## Authors' contributions

MK prepared blood samples, monocyte-dereived dendritic cells, and performed IFN-γ ELISA, KKP participated in drafting the manuscript and data analysis, MOI and MMG performed IHC analysis, LG prepared blood samples, M-LA, EW and X-YW participated in drafting the manuscript and data analysis, HDB coordinated clinical aspects of the study including patient's accrual and participated in drafting the manuscript, MHM conceived the study, designed the experiments, analyzed data and wrote the manuscript. All authors read and approved the final manuscript.
